# The relationship among patient reported outcome measure scores with health care costs and inpatient admission: results from Utah mEVAL and value driven outcomes

**DOI:** 10.1186/s41687-025-00889-y

**Published:** 2025-05-19

**Authors:** Rachel Kroencke, Zoe Gombart, Yue Zhang, Haojia Li, Rachel Hess

**Affiliations:** 1 Department of Internal Medicine, Spencer Fox Eccles School of Medicine, University of Utah, 30 N 1900 E, Room 5R218, Salt Lake City, Utah USA; 2 Department of Internal Medicine, Kaiser Permanente NW, Portland, OR USA; 3 Department of Population Health Sciences, Spencer Fox Eccles School of Medicine, University of Utah, Salt Lake City, Utah USA

**Keywords:** Patient reported outcomes, Healthcare costs, PROMIS

## Abstract

**Background:**

Patient-reported outcomes measures (PROMs) profile patient health status, have been found to be helpful in identifying high health care utilizers, and may be useful in providing targeted interventions to decrease health care costs. In 2013 the University of Utah Health (UU Health) began collecting mental and physical health PROMs using Patient Reported Outcomes Measurement Information System (PROMIS) instruments through a tool called My Evaluation (mEVAL). In 2012 UU Health began cataloguing inpatient and outpatient healthcare-associated costs. The objective of this study was to identify association of poor PROMIS physical function and depression scores with (1) likelihood of inpatient hospitalization and (2) overall inpatient healthcare costs.

**Methodology:**

This study was a retrospective observational cohort study including patients seen at UU Health between 1/2013 and 12/2017 who completed PROMIS instruments at an outpatient visit using the mEVAL platform. PROMIS instruments were completed prior to outpatient visits. The primary outcome was time to incident hospitalization modeled by using the Cox proportional hazards approach. For cost analysis, raw inpatient healthcare costs were fitted using a median regression model. Both results were adjusted.

**Results:**

Of 92,383 people, the average age was 48 (SD 18.6); 57% were female; and 87% identified as non-Hispanic white. A total of 11,909 patients who completed one or both of the mEVAL PROMIS instruments were admitted. The average PROMIS physical function and depression scores were 44.9 and 51.1, respectively. Those with worse physical function scores and worse depression scores were more likely to be hospitalized [HR = 1.77, 95% confidence interval (CI) (1.678, 1.872); HR (95% CI) = 1.149 (1.059, 1.246), respectively]. A physical function score 1.5 SD below the mean was associated with an increased median hospitalization cost of $2496; there was no statistically significant association between depression score 1.5 SD above mean and hospitalization costs.

**Conclusions:**

Poor physical function scores were associated with an increased risk of hospitalization and higher inpatient health costs, while poor depression scores were only associated with increased risk of hospitalization. Future work should examine if improvement in these PROMs alters these metrics.

## Background

Patient reported outcome measures (PROMs) assess a patient’s view of his or her health status. PROMs have many applications ranging from evaluating success of medical treatments to improving health care costs [1, 2]. PROMs have been used to assess stability or progression of chronic disease [3], predict mortality in the setting of new diagnosis [4], and provide a more comprehensive evaluation of responses to medical interventions [5]. PROMs have the potential to identify patient populations that are currently, or more likely to become, high health care utilizers as poor physical function has been associated with increased emergency department visits and hospitalizations [6] and worsening of both mental and physical health has been associated with an increased odds of hospitalization [7]. In this study we sought to determine whether PROMs obtained at outpatient visits could predict likelihood of hospitalization and costs associated with hospitalization to provide a potentially intervenable assessment of hospitalization risk.

In 2004, the National Institutes of Health (NIH) initiated the Patient-Reported Outcomes Measurement Information System (PROMIS) Roadmap project, which incorporated item response theory and computerized adaptive testing to develop modern instruments for assessing PROMs [8]. The PROMIS initiative aimed to create functional, patient-centered, easily comprehended, standardized instruments, absent of rhetorical question items, for improved data collection. As a result, these measures allow more accurate assessment of an individual with fewer questions. The PROMIS instruments are well validated and provide consistent scores within its multiple domains of measurement, such as physical and mental health function [9]. Their brevity and accuracy make them attractive for use in routine clinical care.

In 2013, the University of Utah Health began to implement the standard collection of mental and physical health using PROMIS instruments through a tool called My Evaluation, or mEVAL [5, 10]. Patients complete mEVAL prior to outpatient clinic visits and the scores are integrated into the electronic health record in near real time, providing information regarding the patient health status to the clinician, and contributing to a larger pool of data with the potential for population-based health interventions. Of note, these efforts were suspended during the COVID-19 pandemic due to redeployment of handheld electronic devices towards increasing access to virtual care but are now in the process of resuming. Hospitalization data was only collected for the two years following 2017 as this prevented pandemic related hospitalizations from being included in our analysis.

In these analyses, our objective was to examine the association between PROMs and health care utilization in the inpatient setting. We test the hypotheses that patient’s self-reported physical and mental health are associated with (1) incident hospitalization and (2) overall costs of those hospitalizations.

## Methods

### Study cohort

Patients at the University of Utah who completed a mEVAL PROMIS physical function v1.2 and/or depression v1.0 instrument (https://www.healthmeasures.net/explore-measurement-systems/promis) between January 2013 and December 2017 were included in our analyses. Surveys that were not completed in conjunction with a clinical visit, such as visits with physical therapy, occupational therapy, or speech therapy, and those where the patient was not seen by any provider, were excluded. Data regarding inpatient admissions, patient comorbidities, and patient demographics were extracted from the University of Utah’s Electronic Data Warehouse (EDW), which serves as the data repository for all health system clinical data. This study was deemed non-human subjects by the University of Utah institutional review board (IRB) and exempt from IRB review.

The mEVAL tool was developed by University of Utah Health in 2013 to collect PROMs for use in clinical care. The mEVAL survey employs PROMIS’ computer-adaptive testing to decrease response burden while maintaining sensitivity. Patients can complete mEVAL from home up to one week prior to clinic appointments after receiving an email notification or in the office prior to their visit. There is no incentive for completion of mEVAL surveys.

### Outcomes of interest

Our analysis examined the association between the PROMIS physical function and depression scores and (1) time to first hospitalization within two years of index mEVAL completion and (2) costs of these hospitalizations. We defined the index mEVAL completion as the first time a patient completed a mEVAL survey. Subsequent mEVAL surveys completed were not included in our analysis.

PROMIS scores represent standardized T scores with a mean of 50 for the average of the general population in which they were first studied with a standard deviation of 10. A higher depression PROMIS score indicates greater symptoms of depression reported by the patient while a higher physical function PROMIS score represents higher (or better) physical function.

Costs of inpatient hospitalization were obtained from the University of Utah’s Value Driven Outcomes system (VDO). VDO was developed in 2012 at the University of Utah to determine costs associated with patient care. It provides users with direct clinical care costs associated with each individual clinical encounter, including components such as cost of facilities, healthcare providers, imaging, laboratory, medication, and adjunctive therapy services. The VDO also has the capacity to provide process and outcome measures with the ultimate goal of supporting value-based care [11].

### Other covariates

Other covariates included in these analyses were chosen because of their relationship to either the primary predictors (physical function or depression) or the outcome (incident hospitalization and overall inpatient costs). These included age; sex (male or female); marital status (married, widowed, divorced, single, or other); race/ethnicity (Caucasian/White, American Indian/Alaska Native, Asian, Black/African American, Native Hawaiian and Other Pacific Islander, Hispanic/Latino, or Others); and presence of anxiety, substance, or mood disorder. All confounders were limited to what could be easily extractable from the EDW and are listed in Table 1. As these were secondary analyses of existing data, variables not included in the EDW were not available to include in these analyses. Presence of an anxiety, substance, or mood disorder was evaluated by searching for a pre-determined list of ICD-9 and ICD-10 codes documented within the patient chart. A co-morbidity score was calculated using the van Walraven Elixhauser Score, a modified form of the Elixhauser score [12, 13] with higher scores representing a higher medical complexity. In order to calculate this score diagnostic ICD-9 and ICD-10 codes were pulled for a full 365-day period prior to index mEVAL date for each individual [14].

**Table 1:** Descriptive characteristics of patients with low or high physical function PROMIS scores

Characteristic	Physical function PROMIS score (cutoff point = 29)^a^			
	Total cohort (*N* = 92383)	Low (*N* = 6148)	High (*N* = 86235)	*p*-value
**Age**,** Mean (SD)**	47.9 (18.6)	55.1 (19.2))	47.4 (18.4)	< 0.001
**Female**	53,053 (57%)	3605 (59%)	49,448 (57%)	0.047
**Race**				< 0.001
Caucasian/White	80,117 (87%)	5198 (85%)	74,919 (87%)	
American Indian/Alaska Native	762 (1%)	89 (1%)	673 (1%)	
Asian	1902 (2%)	95 (2%)	1807 (2%)	
Black/African American	1225 (1%)	85 (1%)	1140 (1%)	
Native Hawaiian and Other Pacific Islander	664 (1%)	38 (1%)	626 (1%)	
Others	7713 (8%)	643 (10%)	7070 (8%)	
**Hispanic/Latino**	6478 (7%)	548 (9%)	5930 (7%)	< 0.001
**Marital status**				< 0.001
Married	55,192 (60%)	3267 (53%)	51,925 (60%)	
Divorced	5847 (6%)	662 (11%)	5185 (6%)	
Single	26,202 (28%)	1530 (25%)	24,672 (29%)	
Widowed	3212 (3%)	590 (10%)	2622 (3%)	
Others	1930 (2%)	99 (2%)	1831 (2%)	
**Anxiety disorder**	26,009 (28%)	2371 (39%)	23,638 (27%)	< 0.001
**Substance disorder**	15,845 (17%)	1681 (27%)	14,164 (16%)	< 0.001
**Mood disorder**	29,038 (31%)	2859 (47%)	26,179 (30%)	< 0.001
**Mean van Walraven Elixhauser Score (SD)**	0.9 (3.6)	1.8 (4.7)	0.9 (3.5)	< 0.001
**Admitted**	11,909 (13%)	1578 (26%)	10,331 (12%)	< 0.001

^a^A cut off score of 29 was determined based on 1.5 standard deviations from the mean score for the physical function PROMIS in the general population. A score < 29 was associated with poor self-reported physical function

^b^ Group comparisons were assessed using chi-squared tests for categorical variables and two-sample t-tests for continuous variables, with statistical significance defined as *p* < 0.05. Given the large sample size, many between-group comparisons yield statistically significant p-values (*p* < 0.001). However, not all statistically significant differences reflect clinical meaningful absolute differences. For example, some variables (e.g., race and ethnicity) show small absolute differences despite highly significant p-values, whereas others reflect more substantial differences

### Statistical analysis

#### Incident hospitalization

We used the Cox proportional hazard model [15] to estimate the association of PROMIS physical function and depression scores with time to incident hospitalization up to two years after completion of index mEVAL instrument. Risk of incident hospitalization was reported as an adjusted hazard ratio.

Our primary analysis was performed using mEVAL survey results as a dichotomous independent variable with the cut off 1.5 SD below and above the mean in our sample, for physical function and depression, respectively, with low physical function and high depression scores considered as impaired. The cutoff PROMIS score was 29 for physical function and 65 for depression. These cut off points were chosen based on their ability to separate those with poor physical function and more severe depression from those with only mild or moderate self reports of poor physical function or depression and were based off of the mean scores found in the patient population originally used to develop the PROMIS instrument (https://www.healthmeasures.net/score-and-interpret/interpret-scores/promis/promis-score-cut-points). Additional sensitivity analyses were performed using mEVAL survey results as a continuous independent variable.

#### Costs of care

For patients who had an initial hospitalization during the two years after index mEVAL date and up to current date of data extraction, the costs of hospitalization were extracted from the VDO database. Only costs from the first hospitalization following mEVAL completion were included. Costs from any subsequent hospitalizations or hospitalizations which occurred prior to mEVAL completion were not included in the analysis.

For the cost analysis a median regression model [16] was used with raw costs of hospitalization reported as the outcome. Costs included direct patient costs associated with first hospitalization to occur within 365 days from index mEVAL date. Similar to the analysis performed for hospitalization incidence, mEVAL survey results were used as a dichotomous independent variable with the same cut offs as noted above.

#### Effect modification

An additional analysis was performed to determine effect modification of age and sex on both the incidence of hospitalization and cost outcomes. To differentiate between older and younger patients, the median age was used. This differed between those who completed the physical function mEVAL (48 years) versus the depression mEVAL (56 years).

Analyses were conducted in R Statistical Software version 4.0.3. All data were de-identified by the EDW prior to analysis. All statistical tests were performed at the significant level of 0.05.

## Results

**Fig. 1:**
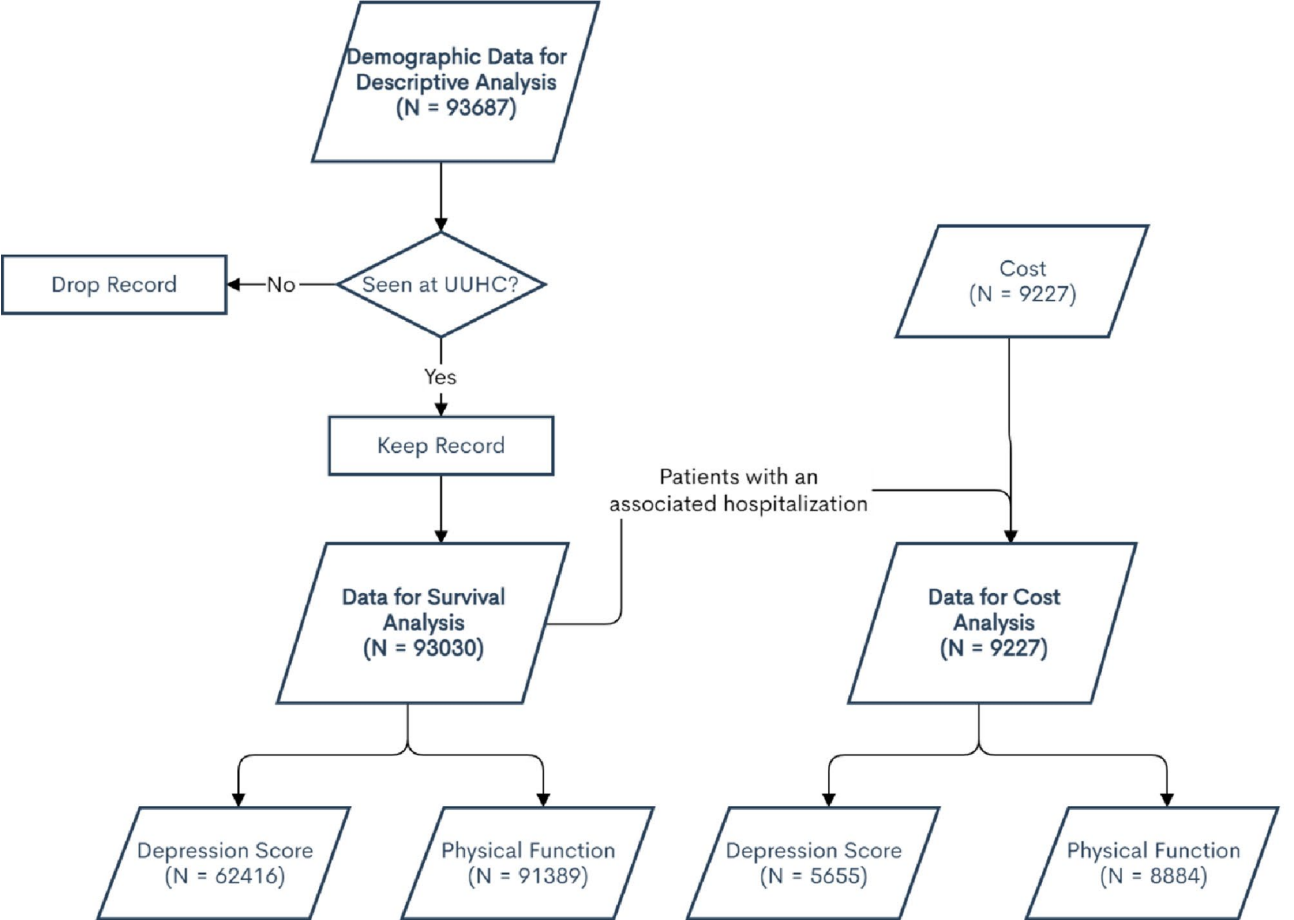
Diagram outlining patient inclusion in analysis for patients seen at University of Utah Health (UUHC)

A total of 93,687 mEVAL surveys were completed between January 2013 and December 2017. Of these, 657 individuals were removed from subsequent analyses due to lack of documented clinical contact associated with the mEVAL completion (i.e., the patient completed the mEVAL survey and then did not attend the associated appointment or the instrument was completed in association with an ancillary service such as physical therapy) resulting in 93,030 individuals included in the analyses. Of these, 92,383 patients completed at least the Physical Function mEVAL survey and 63,344 completed at least the Depression mEVAL survey; 62,040 patients completed both (Fig. 1).

The mean PROMIS Physical Function v1.2 score was 44.9 (standard deviation (SD): 10.5, median 45); 6148 (7%) had low physical function (1.5 SD below the mean, which is a physical function score of 29). Those with low physical function were older than those with high physical function (mean age 55.1 and 47.4, respectively, Table 1). The mean PROMIS Depression v1.0 score was 51 (SD: 9.3; median 51); 4237 (7%) had a high (worse) depression score (1.5 SD above the mean, which is a depression score of 65). Those with higher depression scores were younger than those with lower depression scores (mean age 45.1 and 48.4 respectively, Table 2).

**Table 2:** Descriptive characteristics of patients with low or high depression PROMIS scores

Characteristic	Depression PROMIS score (cutoff point = 65) ^a^			
	Total cohort (*N* = 63344)	Low (*N* = 59107)	High (*N* = 4237)	*p*-value
**Age**,** Mean (SD)**	48.1 (18.5)	48.4 (18.5)	45.1 (17.1)	< 0.001
**Female**	37,733 (60%)	34,962 (59%)	2771 (65%)	< 0.001
**Race**				< 0.001
Caucasian/White	54,932 (87%)	51,373 (87%)	3559 (84%)	
American Indian/Alaska Native	478 (1%)	431 (1%)	47 (1%)	
Asian	1420 (2%)	1356 (2%)	64 (2%)	
Black/African American	768 (1%)	688 (1%)	80 (2%)	
Native Hawaiian and Other Pacific Islander	460 (1%)	423 (1%)	37 (1%)	
Others	5286 (8%)	4836 (8%)	450 (11%)	
**Hispanic/Latino**	4553 (7%)	4131 (7%)	422 (10%)	< 0.001
**Marital status**				< 0.001
Married	38,775 (61%)	36,800 (62%)	1975 (47%)	
Divorced	3907 (6%)	3419 (6%)	488 (12%)	
Single	17,130 (27%)	15,631 (26%)	1499 (35%)	
Widowed	2159 (3%)	2002 (3%)	157 (4%)	
Others	1373 (2%)	1255 (2%)	118 (3%)	
**Anxiety disorder**	19,185 (30%)	16,513 (28%)	2672 (63%)	< 0.001
**Substance disorder**	11,086 (18%)	9693 (16%)	1393 (33%)	< 0.001
**Mood disorder**	21,030 (33%)	17,967 (30%)	3063 (72%)	< 0.001
**Mean van Walraven Elixhauser Score (SD)**	1.2 (4.0)	1.2 (3.9)	0.5 (4.3)	< 0.001
**Admitted**	7527 (12%)	6834 (12%)	693 (16%)	< 0.001

^a^A cut off score of 65 was determined based on 1.5 standard deviations from the mean score for the depression PROMIS in the general population. A score > 65 was associated with greater symptoms of depression

^b^ Group comparisons were assessed using chi-squared tests for categorical variables and two-sample t-tests for continuous variables, with statistical significance defined as *p* < 0.05. Given the large sample size, many between-group comparisons yield statistically significant p-values (*p* < 0.001). However, not all statistically significant differences reflect clinical meaningful absolute differences. For example, some variables (e.g., race and ethnicity) show small absolute differences despite highly significant p-values, whereas others reflect more substantial differences

### Incident hospitalization

Of the 93,030 individuals who completed a mEVAL survey and were seen in clinic at UU Health, 12,049 (13%) had at least one hospital admission (Table 3). The mean age of patients with a hospitalization was 53 years compared to 48 years for the population overall; 61% of hospitalized patients were female (vs. 57% overall), and 87% were non-Hispanic White (vs. 87% overall). Of those with an incident hospitalization, 42% had a documented history of anxiety, 30% a history of substance use disorder, and 48% a history of a mood disorder; average van Walraven Elixhauser Score was 2.2 for those with a hospitalization compared to 0.8 of those without.

**Table 3:** Comparison of hospitalized versus non-hospitalized patients by PROMIS score and other characteristics

Characteristic	Total cohort (*N* = 93687)	Non-hospitalized (*N* = 81638)	Hospitalized (*N* = 12049)	*p*-value
**Physical Function PROMIS score**,** Mean (SD)**	44.9 (10.5)^1^	45.6 (10.3)^2^	39.8 (9.8)^3^	< 0.001
**Depression PROMIS score**,** Mean (SD)**	51.1 (9.3)^4^	50.9 (9.3)^5^	52.6 (9.5)^6^	< 0.001
**Age**,** Mean (SD)**	48.0 (18.6)	47.3 (18.5)	52.7 (18.4)	< 0.001
**Age**				< 0.001
18–34	26,702 (29%)	23,868 (29%)	2834 (24%)	
35–44	14,904 (16%)	13,501 (17%)	1403 (12%)	
45–54	14,299 (15%)	12,738 (16%)	1561 (13%)	
55–64	16,989 (18%)	14,517 (18%)	2472 (21%)	
>= 65	20,792 (22%)	17,013 (21%)	3779 (31%)	
**Female**	53,755 (57%)	46,400 (57%)	7355 (61%)	< 0.001
**Race**				< 0.001
Caucasian/White	81,225 (87%)	70,702 (87%)	10,523 (87%)	
American Indian/Alaska Native	768 (1%)	615 (1%)	153 (1%)	
Asian	1927 (2%)	1712 (2%)	215 (2%)	
Black/African American	1249 (1%)	1054 (1%)	195 (2%)	
Native Hawaiian and Other Pacific Islander	673 (1%)	576 (1%)	97 (1%)	
Others	7845 (8%)	6979 (9%)	866 (7%)	
**Hispanic/Latino**	6582 (7%)	5748 (7%)	834 (7%)	< 0.001
**Marital status**				< 0.001
Married	55,948 (60%)	48,126 (59%)	7822 (65%)	
Divorced	5947 (6%)	4873 (6%)	1074 (9%)	
Single	26,562 (28%)	24,298 (30%)	2264 (19%)	
Widowed	3276 (3%)	2501 (3%)	775 (6%)	
Others	1954 (2%)	1840 (2%)	114 (1%)	
**Anxiety disorder**	26,430 (28%)	21,317 (26%)	5113 (42%)	< 0.001
**Substance disorder**	16,117 (17%)	12,543 (15%)	3574 (30%)	< 0.001
**Mood disorder**	29,577 (32%)	23,823 (29%)	5754 (48%)	< 0.001
**Mean van Walraven Elixhauser Score (SD)**	1.0 (3.6)	0.8 (3.3)	2.2 (5.3)	< 0.001

^1^*N* = 92,383; ^2^*N* = 80,474; ^3^*N* = 11,909; ^4^*N* = 63,344; ^5^*N* = 55,817; ^6^*N* = 7527

^a^ Group comparisons were assessed using chi-squared tests for categorical variables and two-sample t-tests for continuous variables, with statistical significance defined as *p* < 0.05. Given the large sample size, many between-group comparisons yield statistically significant p-values (*p* < 0.001). However, not all statistically significant differences reflect clinical meaningful absolute differences

Scores for physical function were lower for those hospitalized compared to those not hospitalized (mean: 40 vs. 46, *p* < 0.001), and scores for depression were higher for those hospitalized compared to those not (mean: 53 vs. 51, *p* < 0.001). A higher percentage of those with low physical function scores (26% vs. 12%, *p* < 0.001) and a higher percentage of those with high depression scores (16% vs. 12%, *p* < 0.001) were admitted to the hospital.

After controlling for multiple confounders, we found that both those with worse physical function and depression scores were more likely to be admitted to the hospital [HR 1.77, 95% confidence interval (CI) (1.68, 1.87) and HR 1.15, 95% CI (1.06, 1.24), respectively] (Table 4). This association remained significant in secondary analyses using PROMIS scores as continuous independent variables.

**Table 4:** Multivariable association between PROMIS depression and physical function with hospitalizations and costs

Exposure of interest	Outcome of interest	
	Hospitalization	Cost ($)
	HR (95% CI)	Estimated effect (95% CI)
**Depression (by 65)**		
Low		
High	1.15 (1.06,1.24)	833.32 (-204.59,1871.24)
**Physical Function (by 29)**		
High		
Low	1.77 (1.68,1.87)	2502.07 (1770.46,3233.69)

PROMIS: Patient Reported Outcome Measurement Information

System; HR: Hazard Ratio

^a^High depression is indicated by a cutpoint > 65 and low physical function by a cutpoint of < 29

An additional analysis was performed to determine effect modification of age and sex, using PROMIS scores as a dichotomous variable. Different age cut offs were used for the effect modification for physical function versus depression due to different median ages of those who completed each survey. Both older patients, over the age of 48, and younger patients with lower self-reported physical function were more likely to be hospitalized than those with higher self-reported physical function, although the likelihood of admission was much higher in older patients vs. younger patients with low self-reported physical function [Hazard Ratio (HR) 1.90 vs. 1.52, *p* < 0.001]. Sex also had a significantly positive effect modification on the association between low physical function and likelihood of hospitalization, although more so in male patients than in female patients (HR 2.06 vs. 1.60, *p* < 0.001).

Older patients, defined as greater than age 56, with high self-reported depression were more likely to be admitted to the hospital than older patients with lower self-reported depression (HR 1.41, *p* < 0.001). Male patients with high depression scores were more likely to be admitted than those with low depression scores (HR 1.35, *p* = 0.003). The effect modification of female sex was not significant on the association between depression scores and likelihood of admission (Table 5).

**Table 5:** Multivariable association between PROMIS depression and physical function with hospitalizations and costs including interaction between (1) sex or (2) dichotomized age and dichotomized PROMIS depression or physical function scores

		Outcome of interest			
Effect modifier	Exposure of interest	Hospitalization		Cost ($), Median regression	
		Depression (by 65)	Physical function (by 29)	Depression (by 65)	Physical function (by 29)
		**HR (95% CI)**	**HR (95% CI)**	**Estimated effect (95% CI)**	**Estimated effect (95% CI)**
**Sex**	Female	1.05 (0.95,1.16)	1.60 (1.49,1.72)	1340.27 (570.30,2110.24)	3525.47 (2626.13,4424.81)
	Male	1.35 (1.18,1.53)	2.06 (1.90,2.24)	-1185.20 (-2589.00,218.61)	838.95 (-367.11,2045.01)
	**Interaction** **P-value**	0.003	< 0.001	0.003	< 0.001
**Age** ^a^	Low	0.93 (0.83,1.05)	1.52 (1.36,1.69)	1274.71 (-20.32,2569.74)	3914.75 (2811.62,5017.82)
	High	1.41 (1.27,1.57)	1.9 (1.79,2.02)	-1149.66 (-2613.11,313.80)	1864.99 (786.25,2943.72)
	**Interaction** **P-value**	< 0.001	< 0.001	0.045	0.009

PROMIS: Patient Reported Outcome Measurement Information System; HR: Hazard Ratio

^a^Different age cut offs were used for depression (56) versus physical function (48) due to different median ages for those who completed these two instruments

### Costs associated with inpatient care

Among those who were hospitalized, having a PROMIS physical function score of 1.5 SD below the mean was associated with an increased median hospitalization cost of $2502 (*p* < 0.001). PROMIS physical function scores were also significantly associated with hospitalization costs when viewed as a continuous variable ($130, *p* < 0.001). Secondary analysis using a linear regression for transformed direct hospitalization costs showed similar results (data not shown).

Among those who were hospitalized, having a PROMIS depression score of 1.5 SD above mean versus below was associated with a non-significant increase in costs of $833 (*p* < 0.12, Table 4). However, when PROMIS scores were evaluated as a continuous variable, a 1-point increase in the PROMIS depression score was associated with an increased median hospitalization cost of $34 (*p* < 0.001).

Additional analyses were performed to determine effect modification of age and sex on the association between PROMIS physical function scores and inpatient costs. Age and sex were both found to have significant effect modification on the association between dichotomous PROMIS physical function scores and hospitalization costs. Patients older than 48 who reported low physical function cost on average $1864 more than those with higher physical function while younger patients with lower physical function had costs on average $3914 more than those with higher physical function (*p* = 0.009). Female patients with lower reported physical function on average cost $3525 more per hospitalization than female patients with higher physical function (*p* < 0.001). Male sex had no significant effect modification on the association between reported physical function and hospitalization costs.

For depression scores, age did not have a significant effect modification on the association between depression scores and inpatient costs, again when primarily looking at scores as a dichotomous variable. Sex was found to have a significant effect modification on the association between depression scores and inpatient costs (*p* = 0.003). Hospitalizations of female patients with high depression scores cost on average $1340 more than those for female patients with low depression scores. There was no significant effect modification of male sex on the association between depression scores and costs (Table 5).

## Discussion

In these analyses of patients from a single health system, patients with poorer PROMIS physical function and depression scores are more likely to be admitted to the hospital. Additionally, patient reports of physical function are strongly associated with inpatient health costs when controlling for other sociodemographic and medical conditions including substance use, depression, and anxiety. While many studies have evaluated the association of patient reported outcomes in the context of specialty specific procedures, such as orthopedic procedures [5], and individual disease states, such as cancer [4, 17], few have looked at whether patient reports of health from a heterogenous mix of healthcare encounters can predict health care utilization.

In the orthopedic patient population PROMs are more frequently used to assess outcomes from surgical procedures. In the oncology patient population PROMs serve as a prognostic indicator or to assess recovery from treatment such as stem cell transplant. Our study is unique in that it evaluates PROMs ability to predict healthcare utilization for patients of an entire healthcare system, rather than just those receiving treatment for a specific subset of conditions.

Our results add to the available evidence showing PROMs association with a patient’s future healthcare utilization and costs. PROMIS depression and physical function scores could potentially be used to create models to predict a patient’s risk of admission. Similar models have been used in the UK due to the large amount of healthcare data accessible through the NHS [18, 19]. Models with the best predictive value utilize both administrative and clinical data, such as data regarding previous hospitalizations.

Early recognition of these individuals in the outpatient setting may help target interventions aimed at helping to reduce hospitalization and higher health care costs. One prior study attempted to implement this type of intervention by providing patients with real time feedback regarding potential areas of intervention during clinic visits based on their responses to survey data asking about personal health behaviors, medical history, and their responses to a patient reported outcome measure, the health-related quality of life (HRQoL). These surveys were completed prior to the clinic visit. Frequency of patient-initiated discussions regarding these lifestyle risk factors were then measured [20]. Although this study did not assess impact on health care utilization it does represent one mechanism by which patient reported outcomes can be utilized to assess need for medical intervention.

There are several limitations to our analyses. We assume that PROMs are relatively stable and only subject to variation over long periods of time. Although multiple PROMIS scores were collected per individual patient we chose to use only the index, or initial, mEval completed to eliminate any outcome effect from interventions administered in response to PROMIS scores. We also do not factor in potential influence from clinic visits following the initial mEval collection, such as treatments prescribed or interventions performed. Another major limitation is the source of our data. This study only evaluated patients within University of Utah Health and only incorporated hospitalizations and costs occurring at University of Utah Health. Though our sample size is large, it is quite homogenous and younger than the average hospital population, which means our findings are not necessarily translatable to the population of the United States as a whole.

One strength of our study was the collection of mEVALs in both specialty and non-specialty healthcare settings allowing us to broaden the scope from a specialty specific lens. The patients included in this study received health care in both community and academic practices and drew from a large geographic region.

While our study examined only incidence of admission and admission costs associated with inpatient admissions future work could consider whether an association exists between patient reported outcomes and utilization in the outpatient setting. We are also unable to disaggregate the types of health care services that contribute to these higher costs (e.g., length of stay, testing volume).

## Conclusion

Our analysis shows that a patient’s self-reported level of physical function and depression are significantly associated with their risk of hospitalization. Poor physical function is also associated with higher inpatient health costs. PROMs may help identify those at risk for higher costs.

## Data Availability

The datasets generated and/or analysed during the current study are not publicly available due to the proprietary nature of the VDO data but are available from the corresponding author on reasonable request.
